# Equity lessons from a large scale private-sector healthcare intervention in Ghana and Kenya: Results for a multi-year qualitative study

**DOI:** 10.12688/gatesopenres.13142.1

**Published:** 2020-09-14

**Authors:** Dominic Montagu, Lauren Suchman, Charlotte Avery Seefeld

**Affiliations:** 1 Institute for Global Health Sciences, University of California, San Francisco, San Francisco, CA, 94157, USA

**Keywords:** Health Seeking, Private Providers, Equity, Primary Care, Universal Health Coverage

## Abstract

**Background: **The poor fall sick more frequently than the wealthy, and are less likely to seek care when they do.  Private provision in many Low- and Middle-Income Countries makes up half or more of all outpatient care, including among poor paitents.  Understanding the preferences of poor patients which impel them to choose private providers, and how 3 ^rd^ party payment influences these preferences, is important for policy makers considering expansion of national health insurance financing to advance Universal Health Coverage. This paper reports on the results of a qualitative evaluation of the African Health Markets for Equity intiative (AHME), a multi-year initiative in Ghana and Kenya to increase options and improve quality for outpatient services, especially for the poor.

**Methods: **Interviews with patients from private clinics were conducted annually between 2013 and 2018.  Field staff recruited women for exit interviews as they were leaving these clinics. In the final round of data collection (2018), interviewers screened patients for wealth quintile and selected one third of the sample (approximately 10 patients per country) that fell into the two lowest wealth quintiles (Q1 and Q2).  Transcripts were coded using Atlas.ti and coded for analysis using an inductive, thematic approach.

**Results: **We found four primary drivers of patient preferences for private clinics: *convenience; efficiency and predictability*, perceived higher *quality*, and *empowerment* which was derived from greater choice in where to go.

**Conclusions: **Our findings indicate that more options will lead to more opportunities for treatment, and decrease the percentage of those, mostly poor, who become ill and go without care of any kind.  This should be considered as a priority  by policy makers seeking to make the best use of existing national infrastructure and expertise to assure equal health for all.  In this way, private providers offer an opportunity to advance national goals.

## Introduction

The poor fall sick more frequently than the wealthy, and are less likely to seek care when they do. Barriers to care-seeking are complex, but key barriers in Low- and Middle-Income Countries (LMICs) are the compounded effects of limited economic and geographic access. Health systems with significant private sector participation, such as those in Ghana and Kenya, face challenges and opportunities in expanding new financing methods beyond public providers as a way to lower the direct and indirect costs of care-seeking, improve care options, and so advance progress towards University Health Coverage. Further, Private providers are often preferred over public because they are more responsive to patients and are believed to offer higher quality ( Montagu & Goodman, 2016). They outnumber public service delivery points in many countries, particularly in urban areas, and often maintain longer opening hours, shorter wait times, and are closer to access from home or work for many lower-income households ( Morgan *et al.*, 2016).

These aspects of accessibility matter greatly, especially for working class families who may be unable or unwilling to take off the time from paid employment that is often needed for travel and waiting inherent in a public facility consultation; who may value the client-focused interaction and responsiveness that often comes with private ownership; or who may believe, often correctly, that greater attention from private providers is a valid proxy for better care ( Das *et al.*, 2018). In a number of countries, efforts to achieve Universal Health Coverage (UHC) have explicitly included engagement with private providers in order to increase the accessibility of important services ( Chakaya *et al.*, 2009; World Health Organization, 2018). Nonetheless, questions remain about the potential of private sector engagement to serve poor patients without support from a third-party payer. While Social Health Insurance (SHI) schemes have become an increasingly important vehicle to improve health financing for UHC, incomplete knowledge of the rules and expectations associated with financing and delivery, and differences in goals and working styles between government and private partners make inclusion of private providers into SHI a challenge ( Sieverding *et al.,* 2018).

The potential benefits to the health system from private provider inclusion are sufficient that these are challenges worth overcoming, and solutions are being identified ( Montagu & Goodman, 2016; Suchman, 2018). Assuring that those benefits accrue to poor patients as well as middle class and wealthy, requires an understanding of current health-seeking, and of the needs, priorities, and challenges which shape care-seeking decisions for the poor specifically. This paper draws on data from interviews with patients accessing care in urban and peri-urban private clinics in Ghana and Kenya as part of the African Health Markets for Equity intiative (AHME), a multi-year initiative to increase options and improve quality for outpatient services, especially for the poor. Patients were asked about their experiences in past care-seeking for themselves and their families, and the reasons for the choices in where to go for which types of care.

AHME was carried out by a group of NGOs in Ghana and Kenya between 2013 and 2019 with the support of the Bill and Melinda Gates Foundation and the UK Department for International Development ( Kinka *et al.*). Its goal was to increase the number of private providers offering quality maternal and reproductive health services, and in doing so to make quality outpatient care more accessible to low income populations. In parallel to the supply efforts, AHME worked with governments in both Kenya and Ghana to facilitate the inclusion of private providers in the national accreditation schemes in both countries. To increase quality and supply, AHME enrolled existing private providers in a social franchise network; trained and supplied them to facilite provision of a limited number of key public health services, primarily family planning and malaria related, but also including ante-natal care, integrated management of childhood illnesses and pediatric referrals. The providers were supported in step-by-step facility-level quality improvement; loans for investement in facilties; and also assisted in getting accreditation with the national health insurance program (NHI). AHME supported the majority of the aproximately 765 providers enrolled into the Amua and Tunza frachise networks in Kenya, and the 136 providers of the Blue Star franchise in Ghana. In both countries engagement with providers was supplemented by work directly with the NHI’s that aimed to make accreditation and other aspects of work with government regulators more efficient and transparent ( Appleford & Owino, 2017; Appleford, 2019). This included work with government agencies to extend subsidized insurance coverage to low income populations and to enroll them into NHI schemes.

Recent studies in Kenya have shown that the poor seek care less than the wealthy, confirming pediatric care-seeking data from the Demographic Health Surveys ( Chakraborty *et al.*, 2019; National Bureau of Statistics-Kenya & ICF International, 2014). In Kenya, public facilities have a higher proportion of poor clients than do faith-based facilities, which in turn have a higher percentage of poor clients than for-profit or franchised facilities. For-profit and franchised clinics serve quite similar client wealth profiles: unsurprising, as franchise clinics are for-profit clinics, recruited into a franchise network. 

The 2019 Kenya study found that poor clients had a theoretical preference for private providers, but went to public facilities and faith-based clinics because of lower costs ( Chakraborty *et al.*, 2019). Keesara *et al.* found that while poor clients in Kenya valued the convience and efficiency of public facilities, they were less trusting of the clinical quality ( Keesara *et al.*, 2015). Convenience has been highlighted as a primary attraction in Ghana as well ( Ansah *et al.*, 2016; Bamfo & Dogbe, 2017). A 2010 report from the World Bank highlighted regulatory weakness as a cause of poor private-sector clinical quality and the need for accreditation systems to remedy this ( Barnes *et al.*, 2010). 

## Methods

Qualitative interviews with AHME-supported private providers were conducted in 2013, 2015, 2017, and 2018. Interviews with patients were conducted in 2013, 2017, and 2018. Data collection took approximately one month during each round in each country. Field staff traveled to clinics where providers had already been contacted and agreed to participate in an interview. Field staff also recruited women for exit interviews as they were leaving these clinics. In the final round of data collection (2018), interviewers screened patients for wealth quintile and selected one third of the sample (approximately 10 patients per country) that fell into the two lowest wealth quintiles (Q1 and Q2). Interviewers obtained informed consent prior to conducting semi-structured interviews that lasted approximately 60 minutes each. During each round of data collection, providers were asked about their experiences with the AHME interventions and their knowledge of or desire to join any interventions in which they were not currently participating. In 2015, 2017, and 2018 providers also were asked about their perceptions of and experiences with the NHIs. During each round of data collection, patients were asked about their health seeking behavior and during the final two rounds of data collection were asked about their knowledge and experience with the NHIs.

All interviews were recorded using digital recorders in the language the interviewee was most comfortable using. Recordings were translated and transcribed simultaneously by a team of professional transcriptionists who had been trained on key terms. Back-checking for transcription and translation accuracy was done by bi-lingual supervisors in both Ghana and Kenya. All interviews and transcripts were de-identified and audio files encrypted. Audio tapes and files on drives were stored in a secure locked filing cabinet, within a locked office at the IPA research organization offices in each country where interviews took place.

The transcripts were coded using Atlas.ti, version 8. An inductive, thematic approach to coding and analyzing the interviews was used because, particularly in Kenya, there was little existing literature on private providers’ experiences with the NHI’s from which to derive prior theories. An initial coding scheme was created in 2013 based on thematic coding of a sub-set of the interviews from each country and each interview was coded using an open coding approach, in which codes were derived from the data. Common codes were identified across the interviews and grouped into code families and sub-codes. During subsequent rounds of analysis, codes were refined to allow for new priorities in analysis while ensuring continuity across rounds. The codebook was reviewed during each round of analysis to ensure common understanding of codes and consistency in code application.

## Ethical approvals

Ethical approval was provided for each round of data collection by the Ghana Health Service Ethics Review Committee (Protocol #GHS-ERC: 11/05/2013), the Kenya Medical Research Institute (Protocol #Non SSC no. 411), and with “exempt” status from the Institutional Review Board of the University of California San Francisco (Protocol #13-11045). According to the requirements of the local ethical review boards, informed voluntary written consent was obtained from participants in Ghana. Informed verbal consent was obtained from participants before interviews were conducted because of concerns about literacy among low-income household and the desire to obviate the need for participants to provide indentifying information and so minimize patient risk. A script was read emphasisizing patient rights and that participation was voluntary, and verbal consent was received and confirmed by at least two research team members and noted on interview forms before screening questions were asked and interviews begun; this was approved by all IRBs. Interviewees were given the option to withdraw their participation at any time with no consequences for their participation in the AHME interventions. To thank them for their time, participants were given a small gift worth approximately five US dollars, such as a pack of rubber gloves for providers and bar of soap for patients. Consent forms are provided as extended data ( Montagu & Suchman, 2020).

## Results

**Figure 1.**
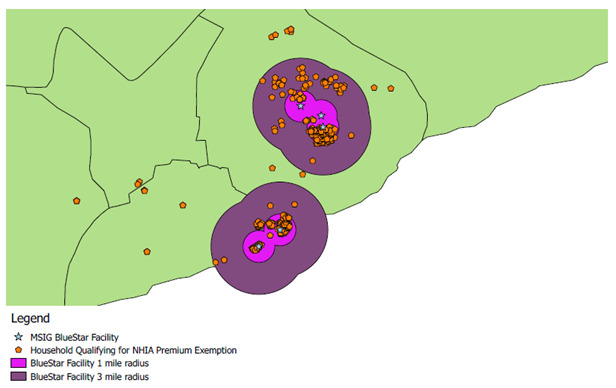
An example of client residence for rural African Health Markets for Equity intiative (AHME) clinics in southern Ghana. (program data replicated with permission).

Programmatic data from the AHME intervention partners in Ghana indicated that clinics in rural or poor areas drew almost all of their clientele from within a 3km distance ( Figure 1). This is reflective of priorities mentioned by patients.

### Prioritizing convenience

There are many more private facilities than public ones in both Kenya and Ghana. Poor clients report that the time it takes to reach a government clinic, and then to wait for services, often puts care out of reach more than any direct fee for care. In rural areas, transportation has added challenges, but even in urban sites, ease of access drives many care-seeking decisions. 

I: Okay, and is this the place where you always come for medication?

R: Yes

I: Why do you like coming here?

R: [Short pause] because it’s nearby, it’s not far, and [short pause], it’s good for everyone.

                                                                                                                                                         (Patient at an Amua Facility, Rift Valley, Kenya, Q1)

So you know it depends. You see here is near home so you see you come here, eh.

                                                                                                                                                         (Patient at a Tunza Facility, Rift Valley, Kenya, Q2)

### Prioritizing efficiency and predictability

Private providers are more convenient for many, and they are also – critically – often more efficient. When time equals money, for the wealthy or the working poor, the cost of a day lost waiting in lines, or a day of lost income, can make private providers more attractive than the less efficient public sector despite the out-of-pocket expense. After explaining that she would have to pay only 20 shillings (0.20 USD) as an informal charge for services at a government center, and 100 shillings (1 USD) at a private clinic, one mother in Kenya said that she nevertheless opted for the private site:

[At the government site] if you come for the family planning you pay 20 shillings for the number, then you get to be treated free, isn’t it?… Yes, if we compare I see they are so much because it wastes my time the whole day that I can go do a job and be paid 500 shillings. So I feel 100 [here at a private clinic] is less in the end.

                                                                                                                                                         (Patient at a Tunza Facility, Eastern, Kenya, Q1)

The charge she mentions was an ‘informal’ fee for a number to enter into the service line. This complaint that even government free services are never really free was common, and accepted as simply the normal way that the world works. 

You know in government people pay a lot of money, and here [at a private clinic] they take a little… even if you come in with 200 they just take. 

                                                                                                                                                         (Patient at a Tunza Facility, Nyanza, Kenya, Q1)

Most challenging of all, for the poor, is that even if one pays the informal fees at government facilities, there are still a host of extra costs for care that aren’t covered by either free services for the poor, or by national health insurance. But at least the consultation is, sometimes, free if one has no money to pay.

Now at the public hospital you won’t be charged but you know it is the medicine that are missing, but you won’t be charged.

                                                                                                                                                         (Patient at a Tunza Facility, Rift Valley, Kenya, Q2)

Of course, some respondents highlighted that even if one *does* pay money to a government site, there may still be extra fees. And if one goes without enough money, there will *definitely* be extra fees or else some things, like free medicines, will not be available.

… when you go to the government they even take 1500 [Shillings], and if you go with 500 they can’t give you all the medicine.

                                                                                                                                                         (Patient at a Tunza Facility, Nyanza, Kenya, Q1)

Negotiations and uncertainty are stressful as well as costly and time-consuming. As a result, for those who can afford it, the predictability of care in the private sector is also an attraction.

…in government there is no payments done and you can go there without money and you won’t get drugs. You come here [to a private clinic] you pay and go home with medicine.

                                                                                                                                                         (Patient at a Tunza Facility, Eastern, Kenya, Q2)

Even for the poor, these benefits may be significant enough that it is worth finding a way to make the costs of private care viable. As one might imagine, the market is ready to help: in both Kenya and Ghana providers are reportedly eager to work with clients to make their services accessible. As one poor mother in Kenya said when asked how much she paid for treatment in a private clinic:

Interviewer: 300 Shillings, what do you think about that money, is it expensive or less?

Respondent: It is less; sometimes he can’t ask you to pay everything but can accept instalments. Even when you have no money, he can still treat the child for you.

                                                                                                                                                         (Patient at an Amua Facility, Eastern, Kenya Q1)

Even higher-income clients reported paying for services on credit or negotiating price when they visited a private health facility. This suggests that costs may be prohibitively expensive regardless of socioeconomic status and that a third party payer is key to making health services more accessible for all.

### Prioritizing quality

Patients are often not able to express in words why they believe the quality is better at private providers than at public facilities, but they do think that and believe that availability of medicines, and outcomes, prove them right. As two patients said when asked why they choose private for treatment for their children:

Because when I come here, the child gets well.

                                                                                                                                                         (Patient at an Amua Facility, Rift Valley, Kenya, Q1)

The cost is a bit higher than the government hospital but because you also want you welfare, you continue coming because you know that at the end of the day even if you pay high you are being offered a good service.

                                                                                                                                                         (Patient at a BlueStar Facility, Central, Ghana)

### 3 ^rd^ party payer empowers patients

Ghanaian patients suggested that having NHI coverage gave them more options when choosing a provider: with insurance, private clinics and hospitals at different levels of the health system become viable options. 

Well, some of the eh the private clinics they accept this thing, the health insurance. It covers some parts and the hospital too, the government hospital too, that one too is the same thing, so [we can go to] any of them.

                                                                                                                                                         (Patient at a BlueStar Facility, Ashanti, Ghana, Q2)

Linkages into the public sector, and affordability because of insurance, become significant motivations in selecting a provider:

There are at times I don’t have money to visit clinics that don’t accept health insurance that’s the reason why I don’t visit such clinics.

                                                                                                                                                         (Patient at a BlueStar Facility, Volta, Ghana, Q2)

People also appreciate that third-party payers helps defray costs. Patients in Ghana mentioned that people would die without insurance.

It has been beneficial to us because we would have spent a lot of money, more than one million [old Ghana cedis] (18USD) if you don’t have the NHIS. But if you have it you won’t spend anything.

                                                                                                                                                         (Patient at a BlueStar Facility, Volta, Ghana, Q1)

## Discussion

In both countries providers and patients emphasized the increased access and choices that came together with enrolling into NHI. For providers this meant new patients. For patients, it meant more options, and perhaps easier access to the nearer, and more responsive, providers they preferred. 

NHI funding in both Kenya and Ghana is expanding, and coverage of the informal workers and the poor in both countries remains low. Nevertheless, the lessons from our research is that at all income levels healthcare costs, and the opportunity costs of seeking and waiting for care, are a burden. Choices are determined by many factors, including perceived quality, wait-time, and costs. And so the more that costs can be reduced, the more that choices available to the wealthy can be shared across all citizens. Choices matter greatly, as DHS data shows that in Kenya, Ghana and many other countries, the poor often forgo care of any sort when ill ( Bradley *et al.*, 2017).

Our findings show four primary drivers of patient preferences which indicate the value placed on the private sector, and the existing and potentially larger future impact of expanding NHI funding for privately provided primary care. *Convenience*, both geographic and opening hours. *Efficiency and Predictability* matters both for the wealthy who buy their way out of long lines in government clinics, and for the poor, who cannot afford to wait for free care if that might mean losing a day of pay. *Quality*, perceived or real: patients often associate the care they get in private clinics with better outcomes. *Choice and empowerment*, to receive the same care, at the same out-of-pocket cost, regardless of where one goes. 

## Conclusion

Our findings did not indicate a universal belief that private providers in Ghana and Kenya are better, or smarter, or less expensive, or always preferable to government facilities. But for some clients the private sector serves their needs and preferences best. When national insurance is able to reduce the financial barriers to accessing care, the resulting increase in choice is welcomed gladly. Universal Health Coverage efforts offer a special opportunity to increase financing for primary care that is ‘ownership agnostic’, and so to provide more accessible and more diverse choices of where to go when ill; choices in care-seeking which are valued by both wealthy and poor clients in our survey. The provision of financial support through NHIs may also provide governments with new opportunitites to increase the effectiveness of referral and regulatory systems, and apply controls on quality, un-authorized payment demands, and patient mis-treatment. Concerns about these issues – in both public and private settings - are a barrier to care seeking for many. 

Findings from our research indicate that more options will lead to more opportunities for treatment, and decrease the percentage of those, mostly poor, who become ill and go without care of any kind. This is at the heart of advancing Universal Health Coverage, and should be considered as a priority by policy makers seeking to make the best use of existing national infrastructure and expertise to assure equal health for all. In this way, private providers offer an opportunity to advance national goals.

## Data availability

### Underlying data

The study Consent forms preclude sharing of interview transcripts beyond immediate research members. Attempts to revise these to allow de-identified transcripts from later survey rounds to be shared were not permitted by the Kenyan Institutional Review Board. The Review Board of the University of California, San Francisco determined that the wording of the Consent form from 2011 prohibits transcripts and data within analysis software from being shared outside of the research team. Our Consent Form is provided as extended data ( Montagu & Suchman, 2020).

### Extended data

DRYAD: Qualitative survey instruments for a study on equity from a large-scale private-sector healthcare intervention in Ghana and Kenya: the African Health Markets for Equity (AHME) study. https://www.doi.org/10.7272/Q6FX77NG ( Montagu & Suchman, 2020)

This project contains the following extended data:

- Consent_Form_Kenya_Verbal.pdf (IRB approved form for Verbal Consent in Kenya)
- Consent_Form_Written_and_Verbal.pdf (IRB approved form for Consent, both Kenya and Ghana. Includes option for verbal consent)
- Guide_FGD_Community_Member_Female_2013.docx (Guide for women-only focus group survey)
- Guide_FGD_Community_Member_Male_2013.docx (Guide for men-only focus group survey)
- Guide_IDI_Franchise_Patient_2013.docx 92013 patient interview guide)
- Guide_IDI_Franchise_Patient_2016.docx (2016 patient interview guide)
- Guide_IDI_Franchise_Patient_2017.docx (2017 patient interview guide)
- Guide_IDI_Franchise_Patient_Ghana_2018.docx (2018 patient interview guide, Ghana)
- Guide_IDI_Franchise_Provider_2013.docx (2013 provider interview guide)
- Guide_IDI_Franchise_Provider_Kenya_2017.docx (2017 provider interview guide, Kenya)
- Guide_IDI_Franchise_Provider_Kenya_2018.docx (2018 provider interview guide, Kenya)
- Guide_IDI_Franchise_Provider__Ghana_2018.docx (2018 provider interview guide, Ghana)
- Guide_IDI_Implementer_2019.docx (2019 implementer interview guide)
- Guide_IDI_NHI_2019.docx (2019 interview guide, Nat. Health Insurance staff)
- Guide_IDI_Non-Franchise_Provider_Kenya_2017.docx (2017 provider interview guide, non-Franchise, Kenya)
- Guide_IDI_Non-Franchise_Provider_Kenya_2018.docx (2018 provider interview guide, non-Franchise, Kenya)
- Guide_IDI_Stakeholders_2013.docx (2013 stakeholder interview guide)
- Guide_IDI_Stakeholders_global_2019.docx (2019 international stakeholder interview guide)

Data are available under the terms of the Creative Commons Zero "No rights reserved" data waiver (CC0 1.0 Public domain dedication).
