# Supplementary material for: Equity lessons from a large scale private-sector healthcare intervention in Ghana and Kenya: Results for a multi-year qualitative study
Source: Gates Open Res. 2020 Sep 14;4:129. [Version 1] doi: 10.12688/gatesopenres.13142.1 (PMC7520554; doi:10.12688/gatesopenres.13142.1)
Supplement: Peer review history

# Peer review: Reviewer response for version 1

**DOI:** 10.21956/gatesopenres.14331.r29617

**Published:** 2020-11-25

**Authors:** Guy Stallworthy

**Affiliations:** 1 Bill & Melinda Gates Foundation, Seattle, WA, USA

This is a valuable contribution to the evidence on the motivations of healthcare consumers in LMIC as they make choices between different sources of healthcare services. It highlights the importance of patients' time and the ways in which nominally "free" care may be more costly. The opportunity to gather and analyze consistent data from multiple sites in two countries over five years is rare.

 While the descriptions of patient motivations were clear, they could have been labelled a little more rigorously: "efficiency" (which here seems to refer to time spent) and "predictability" were somehow combined, and "empowerment" seems to refer to choice. The labels matter as they could influence the framing of key messages for the reader. In a scientific paper one might expect a less conversational style and more conventional grammar. The analysis might have benefitted from presentation of the findings from the interviews that were conducted with healthcare providers. It would also have been interesting to explore the motivations of male clients.

Is the work clearly and accurately presented and does it cite the current literature?

Yes

If applicable, is the statistical analysis and its interpretation appropriate?

Not applicable

Are all the source data underlying the results available to ensure full reproducibility?

Yes

Is the study design appropriate and is the work technically sound?

Yes

Are the conclusions drawn adequately supported by the results?

Partly

Are sufficient details of methods and analysis provided to allow replication by others?

Yes

Reviewer Expertise:

Engagement of private healthcare providers for primary care in low- and middle-income countries.

I confirm that I have read this submission and believe that I have an appropriate level of expertise to confirm that it is of an acceptable scientific standard.

# Peer review: Reviewer response for version 1

**DOI:** 10.21956/gatesopenres.14331.r29582

**Published:** 2020-09-25

**Authors:** Luke Boddam-Whetham

**Affiliations:** 1 Palladium, London, UK; 2 Marie Stopes International, London, UK

This is a succinctly written piece highlighting clearly some interesting findings around client motivations for using private sector primary health care providers. It shows that cost, in terms of a fee paid for a service or medication is only one factor in decision making about where to access services - for clients poor and non-poor alike.

 If the data is available it would be interesting to know if any of the four drivers of patient preferences had greater influence on decision making and if this differed between poor and non-poor interviewees.

 Given that clinical quality in private facilities is rarely found to be better than in public facilities it is interesting to note the client perception that outcomes are better and I wonder if the authors explored this perception further.  

Is the work clearly and accurately presented and does it cite the current literature?

Yes

If applicable, is the statistical analysis and its interpretation appropriate?

Not applicable

Are all the source data underlying the results available to ensure full reproducibility?

No source data required

Is the study design appropriate and is the work technically sound?

Yes

Are the conclusions drawn adequately supported by the results?

Yes

Are sufficient details of methods and analysis provided to allow replication by others?

Yes

Reviewer Expertise:

Private sector engagement in health systems, health financing.

I confirm that I have read this submission and believe that I have an appropriate level of expertise to confirm that it is of an acceptable scientific standard.

# Peer review: Reviewer response for version 1

**DOI:** 10.21956/gatesopenres.14331.r29586

**Published:** 2020-09-21

**Authors:** Stefan Nachuk

**Affiliations:** 1 Bill & Melinda Gates Foundation, New Delhi, Delhi, India

It's a nice paper that articulates why the poor make the choices that they do in terms of health seeking behavior, and why convenience, perceived quality, and opportunity cost (of lost time) may pull poor clients towards private providers. Without adding a huge amount to the text, it would be helpful to add a few things:

- The fact base - at least a cursory description of NHIA Ghana and NHIF Kenya. Percentage covered, sources of revenues, services covered (both quite generous on paper). Something on the current split in terms of amounts of funds that go to private/public providers overall. 
- The discussion - one senses that the interviews surfaced quite a bit more information, and if there were insights regarding whether most interviewees used both public and private facilities (implied, but not quite clear) and in which cases they went public. Is it only when people are flat broke? The term quality appears to refer to being treated quickly and nicely, and receiving medicines? Is this the case? Deepening the discussion on opportunity costs of going public would be very helpful, as you hint that private charges more, but may be cheaper for the client when lost time is accounted for. Is this the case for most clients interviewed? All clients? How much information did clients have regarding their health insurance benefits?  

Is the work clearly and accurately presented and does it cite the current literature?

Yes

If applicable, is the statistical analysis and its interpretation appropriate?

Not applicable

Are all the source data underlying the results available to ensure full reproducibility?

Yes

Is the study design appropriate and is the work technically sound?

Yes

Are the conclusions drawn adequately supported by the results?

Yes

Are sufficient details of methods and analysis provided to allow replication by others?

Yes

Reviewer Expertise:

Health systems, health financing

I confirm that I have read this submission and believe that I have an appropriate level of expertise to confirm that it is of an acceptable scientific standard.
